# A Review on Biomarker‐Enhanced Machine Learning for Early Diagnosis and Outcome Prediction in Ovarian Cancer Management

**DOI:** 10.1002/cam4.71224

**Published:** 2025-09-10

**Authors:** Somayyeh Hormaty, Anwar Nather Seiwan, Bushra H. Rasheed, Hanieh Parvaz, Ali Gharahzadeh, Hamid Ghaznavi

**Affiliations:** ^1^ Stem Cell and Tissue Engineering Department Istinye University Istanbul Turkey; ^2^ Department of Chemistry and Polymer Science Istanbul Technical University Istanbul Turkey; ^3^ Department of Biology, College of Science Basrah University Basrah Iraq; ^4^ College of Education Ibn Al‐Haytham University of Baghdad Baghdad Iraq; ^5^ Department of Computer Engineering, Faculty of Engineering Shahid Chamran University of Ahvaz Ahvaz Iran; ^6^ Department of Operating Room, Torbat Jam Faculty of Medical Sciences Torbat Jam Iran; ^7^ Department of Computer Engineering, Social and Biological Network Analysis Laboratory University of Kurdistan Sanandaj Iran

**Keywords:** early detection, machine learning, ovarian cancer, precision medicine, prognosis biomarkers

## Abstract

**Background:**

Ovarian cancer (OC) remains the most lethal gynecological malignancy, largely due to its late‐stage diagnosis and nonspecific early symptoms. Advances in biomarker identification and machine learning offer promising avenues for improving early detection and prognosis. This review evaluates the role of biomarker‐driven ML models in enhancing the early detection, risk stratification, and treatment planning of OC.

**Methods:**

We analyzed literature spanning clinical, biomarker, and ML studies, emphasizing key diagnostic and prognostic biomarkers (e.g., CA‐125, HE4) and ML techniques (e.g., Random Forest, XGBoost, Neural Networks). The review synthesizes findings from 17 investigations that integrate multi‐modal data, including tumor markers, inflammatory, metabolic, and hematologic parameters, to assess ML model performance.

**Findings:**

Biomarker‐driven ML models significantly outperform traditional statistical methods, achieving AUC values exceeding 0.90 in diagnosing OC and distinguishing malignant from benign tumors. Ensemble methods (e.g., Random Forest, XGBoost) and deep learning approaches (e.g., RNNs) excel in classification accuracy (up to 99.82%), survival prediction (AUC up to 0.866), and treatment response forecasting. Combining CA‐125 and HE4 with additional markers like CRP and NLR enhances specificity and sensitivity. However, limitations such as small sample sizes, lack of external validation, and exclusion of imaging/genomic data hinder clinical adoption.

**Conclusion:**

Biomarker‐driven ML represents a transformative approach for OC management, improving diagnostic precision and personalized care. Future research should prioritize multi‐center validation, multi‐omics integration, and explainable AI to overcome current challenges and enable real‐world implementation, potentially reducing OC mortality through earlier detection and optimized treatment.

## 1 Introduction

Ovarian cancer (OC) is the fifth most common cause of cancer‐related death among women and the most lethal gynecologic malignancy [1]. Most ovarian cancer cases are diagnosed in an advanced stage, which substantially increases the risk of recurrence and early death [2]. Epithelial ovarian cancer (EOC) which makes up nearly 90% of all OC cases, is particularly challenging to detect at an early stage, leading to poor prognosis and high recurrence rates [3, 4]. OC is usually diagnosed at advanced stages, with a 5‐year survival rate of about 30%. Early detection significantly improves outcomes—survival reaches 84% for localized disease but drops to 32% for distant disease. However, only ~5% of high‐grade serous cases are found early, highlighting the need for better early detection methods [5].

The diagnosis of ovarian cancer (OC) relies on a combination of clinical assessment, biomarker evaluation, and imaging techniques. Conventional imaging modalities—including ultrasound (US), multidetector computed tomography (MDCT), magnetic resonance imaging (MRI), and fluorodeoxyglucose positron emission tomography/computed tomography (FDG PET/CT)—play a crucial role in assessing tumor morphology, staging, and metastatic spread [6]. Although MRI demonstrates the highest sensitivity and FDG PET/CT the highest specificity, no significant differences in overall diagnostic performance were observed among the three techniques. Therefore, MDCT, due to its speed, cost‐effectiveness, and widespread availability, remains the preferred first‐line imaging method when a stand‐alone modality is required. In cases where MDCT results are inconclusive, MRI or PET/CT may provide additional diagnostic clarity. Notably, whole‐body FDG PET/CT may offer superior accuracy in detecting supradiaphragmatic metastases [7]. A recent meta‐analysis further supports this, showing that [^18^F]FDG PET/CT offers higher sensitivity (94%) compared to MRI (87%) for initial ovarian cancer diagnosis, with comparable specificity, highlighting its potential value when more precise detection is required [8].

Cancer antigen 125 (CA‐125), also known as carbohydrate antigen 125, has long been the most widely used biomarker for detecting ovarian cancer, particularly in monitoring treatment response and recurrence [9]. Although CA‐125 is widely used, it is not very specific to ovarian cancer. Its levels can rise in many other cancers like endometrial, pancreatic, or breast cancer, as well as in non‐cancerous conditions such as endometriosis, liver disease, or even during pregnancy and the menstrual cycle [10, 11, 12]. This makes it harder to rely on CA‐125 alone for accurately diagnosing ovarian cancer. To improve diagnostic accuracy, researchers have explored complementary biomarkers, including Human Epididymis Protein 4 (HE4), CA72‐4, mesothelin, transthyretin (TTR), apolipoprotein A‐I (ApoA1), kallikrein, and osteopontin (OPN). Additionally, emerging prognostic biomarkers such as vascular endothelial growth factor (VEGF), prostasin (PSN), transferrin, and bikunin have shown promise in predicting tumor progression and treatment outcomes [13, 14, 15, 16, 17, 18]. However, a meta‐analysis suggests that human epididymis protein 4 (HE4) may offer superior diagnostic accuracy, with higher sensitivity and specificity in differentiating malignant from benign gynecological conditions [19]. Multi‐biomarker panels and risk assessment models, such as the Risk of Ovarian Malignancy Algorithm (ROMA), which integrates CA‐125 and HE4, have demonstrated improved specificity in ovarian cancer diagnosis, particularly in distinguishing malignant tumors from benign ovarian cancer [20, 21]. The UK Collaborative Trial of Ovarian Cancer Screening (UKCTOCS) trial evaluated whether ovarian cancer screening could reduce mortality in over 200,000 women using either multimodal screening (MMS: CA‐125 + ultrasound) or ultrasound alone (USS). After long‐term follow‐up, neither method significantly reduced ovarian/tubal cancer deaths. MMS slightly increased early‐stage detection and reduced stage IV cases, but this modest stage shift was insufficient to impact mortality. The study highlights the limitations of current screening approaches and the need for more effective early detection strategies [22].

**FIGURE 1:**
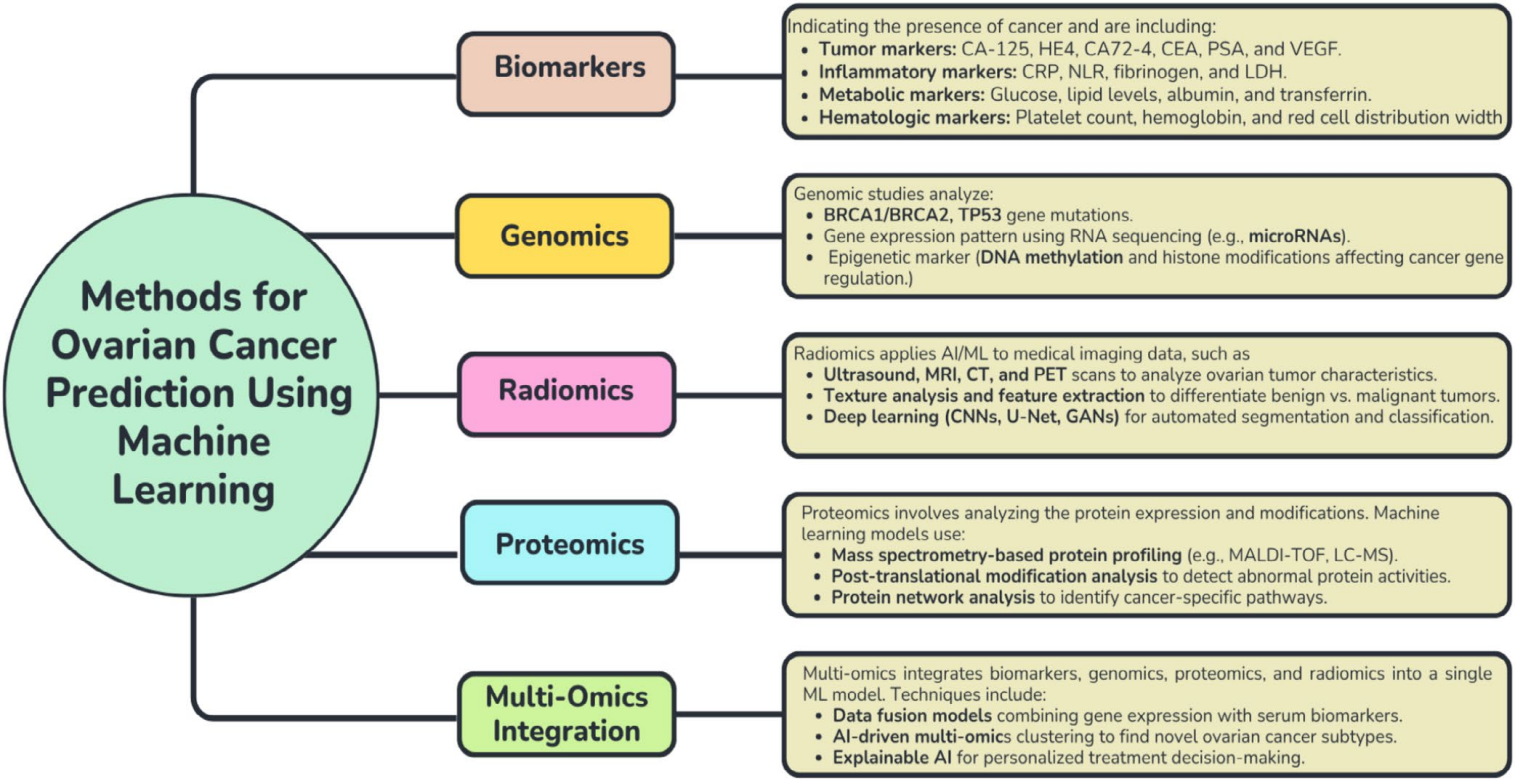
Methods for ovarian cancer prediction using machine learning.

Recent developments in machine learning (ML) and artificial intelligence (AI) have transformed cancer research by facilitating early detection, enhancing diagnostic precision, and supporting personalized treatment approaches [23, 24, 25, 26]. ML algorithms can analyze large, complex datasets and uncover hidden patterns in clinical, molecular, and imaging data [25]. Studies have demonstrated that ML models such as random forests (RF), support vector machines (SVM), and gradient boosting machines (XGBoost) can outperform traditional statistical methods in ovarian cancer prediction [27, 28]. Beyond traditional biomarker‐based models, multi‐omics integration, which combines genomics, proteomics, radiomics, and clinical variables, has emerged as a powerful tool for precision oncology [29]. Figure 1 illustrates various methods for improving ovarian cancer prediction using different data sources and machine learning. By integrating tumor marker data with gene expression profiles, metabolic signatures, and radiomic imaging features, AI‐driven approaches have the potential to develop highly personalized OC risk prediction models [30]. However, despite advancements in genomics [31], proteomics [32], radiomics [33], and multi‐omics integration [34], biomarker‐based ML models remain the most practical and clinically feasible approach for ovarian cancer prediction. While these alternative methods offer valuable insights, they often require specialized infrastructure, high computational resources, and large, well‐annotated datasets, limiting their immediate clinical applicability [35].

This review prioritizes biomarker‐driven ML models due to their non‐invasive nature, cost‐effectiveness, and widespread clinical adoption [36]. Although future advancements in multi‐omics AI models may further enhance ovarian cancer prediction, biomarker‐based ML approaches currently offer the most scalable and readily implementable solution for improving early detection, risk stratification, and treatment decision‐making. By focusing on biomarker‐integrated ML models, this review highlights a clinically viable and actionable strategy for enhancing ovarian cancer prediction. As AI‐driven healthcare continues to evolve, the integration of machine learning with validated biomarkers holds great promise for earlier diagnosis, optimized treatment planning, and improved patient outcomes in ovarian cancer management.

## 2 Biomarkers in Ovarian Cancer Prediction

A cancer biomarker is a measurable characteristic that indicates cancer risk, presence, or patient prognosis. These biomarkers can be molecular, cellular, physiological, or derived from imaging techniques [37]. Personalized medicine increasingly depends on biomarkers to identify ideal candidates for specific treatments. These biomarkers inform prognosis, predict treatment response, and optimize dosage, ultimately guiding therapeutic choices [38]. Although tumor tissue has historically been the primary source for cancer biomarker analysis, the field is increasingly exploring non‐invasive liquid biopsies [37, 39]. Biopsies done from blood, urine, or stool in this manner are some of the least invasive methods to look for cancer. Other types of samples such as sputum, cerebrospinal fluid, and other body fluids can also help in diagnosing but are used only occasionally [39, 40, 41]. Among more than fifteen ovarian cancer‐related biomarkers identified to date, the most widely studied include CA‐125, HE4, kallikreins, prostasin (PSN), transthyretin (TTR), transferrin, vascular endothelial growth factor (VEGF), apolipoprotein A‐I (ApoA‐I), and osteopontin (OPN) [42]. A summary of these diagnostic biomarkers is provided in Table 1 [42].

**TABLE 1:** Selection of diagnostic and prognostic markers usually screened in the clinics.

Biomarker	Full name	Source	Features	Clinical role	Diagnostic marker?	Prognostic marker?
CA‐125	Carbohydrate antigen 125	Serum	Highly present in 80% of late‐stage epithelial OC	Detection and diagnosis	Yes	Yes
HE4	Human epididymis protein 4	Serum	Expressed in endometrioid and serous OC	Detection and diagnosis	Yes	No
KLK	Kallikrein	Serum	Upregulated in OC with poor prognosis and chemoresistance	Detection and diagnosis	Yes	No
PSN	Prostasin	Serum	Expression levels > 100 in epithelial and stromal OC vs. normal condition	Detection and diagnosis	Yes	No
TTR	Transthyretin	Serum	Low levels in OC	Detection and diagnosis	Yes	No
TF	Transferrin	Serum	Low levels in OC	Detection and diagnosis	Yes	No
VEGF	Vascular endothelial growth factor	Serum	Direct correlation with OC	Detection and diagnosis	Yes	Yes
Bikunin	—	Serum	High levels related to favorable prognosis	Prognosis and survival rate	No	Yes
CKB	Creatine kinase B	Serum	Highly expressed in early tumoral phases	Prognosis and survival rate	Yes	Yes
ApoA‐I	Apolipoprotein A‐I	Plasma	Low levels in OC	Detection and diagnosis	Yes	No
OPN	Osteopontin	Plasma	Highly expressed in OC	Progression and metastasis	Yes	Yes

*Cancer antigen 125 (CA‐125)* is a protein encoded by the MUC16 gene which is a widely used serum biomarker for OC [43]. While elevated levels are seen in up to 80% of women with late‐stage epithelial ovarian cancer, its utility for early detection is hampered by the fact that only about half of early‐stage cases show increased CA125 [44, 45]. Its specificity is also limited due to elevations in benign conditions like endometriosis and liver cirrhosis [46]. The UKCTOCS demonstrated that longitudinal CA‐125 measurements achieved a 24.5% reduction in stage IV disease but no significant mortality benefit, highlighting its limitations in population screening [22]. The Risk of Malignancy Index (RMI), combining CA‐125, menopausal status, and ultrasound, improves diagnostic accuracy (sensitivity 87%, specificity 97%) [47].

*Cancer Antigen 19‐9 (CA 19–9)* is primarily used for pancreatic and gastrointestinal cancers but is elevated in some OC cases, particularly mucinous tumors, aiding differentiation of benign from malignant tumors when CA‐125 is normal [48, 49].

*Carcinoembryonic Antigen (CEA)* is a tumor marker that can be elevated in various types of cancer, including colonic (colorectal) and ovarian carcinoma [50, 51]. It is less specific, but when combined with CA‐125, CA 19–9, and tumor size, enhances the diagnosis of mucinous OC [52].

*Human epididymis protein 4 (HE4)*, encoded by WFDC2 [53], is a highly specific OC biomarker, expressed in most serous and endometrioid carcinomas but not in normal ovarian tissue [54, 55]. HE4 outperforms CA‐125 in specificity, especially in premenopausal women, and is integral to ROMA, which improves risk stratification [56].

*Kallikreins (KLKs)* are a family of serine proteases that show elevated levels in OC [57]. KLK6 and KLK7 have shown promise as biomarkers for early detection and diagnosis of OC, especially the serous and papillary serous subtypes [58]. **KLKs** are under investigation for diagnosing tubo‐ovarian abscesses, though further validation is needed [59].

*Human Prostasin (PSN)* is a trypsin‐like protease and plays a crucial role in activating epithelial sodium channels and inhibiting the invasion of prostate and breast cancer cells [60]. PSN is elevated in OC and shows high sensitivity and specificity for early detection, complementing CA‐125 and HE4, with post‐surgical declines indicating prognostic potential [61, 62].

*Vascular Endothelial Growth Factor (VEGF)* a crucial protein for angiogenesis, is essential for ovarian tumor growth [63]. High VEGF expression in ovarian cancer correlates with worse prognosis and reduced survival [64]. It also contributes to immune evasion by increasing myeloid‐derived suppressor cells (MDSCs), which inhibit anti‐tumor immune responses [65]. Studies have shown that elevated VEGF—especially in patients with residual disease—correlates with worse outcomes and is positively linked with malondialdehyde, suggesting a connection between angiogenesis and oxidative stress, making VEGF a key prognostic biomarker in ovarian cancer [66].

*Transthyretin (TTR)* is an endogenous serum protein mainly produced in the liver [67]. It plays a role in hormone transport and has been used as a marker of nutritional and inflammatory status [68, 69]. Studies show TTR offers higher sensitivity than CA‐125 in early‐stage ovarian cancer, suggesting its potential as a complementary biomarker for early detection [70].

*Transferrin (TF)* plays a key role in iron transport by binding two ferric iron atoms along with an anion, usually bicarbonate [71]. Studies suggest altered transferrin levels in ovarian cancer. For instance, one study observed a twofold decrease in grade 3 patients compared to healthy women [72]. While TF alone may have limited diagnostic value, its effectiveness improves when combined with other markers such as CA‐125, apolipoprotein A‐I, and transthyretin for early ovarian cancer detection [73, 74].

*Apolipoprotein A‐I (ApoA‐I)* is a component of high‐density lipoprotein (HDL) found in plasma [75]. It shows promising potential as a biomarker for early detection of ovarian cancer—especially when used alongside TTR and TF [76, 77]. Beyond its diagnostic value, ApoA‐I might also contribute to cancer prevention and therapy, although more research is needed to fully understand its underlying anti‐tumor effects [78].

*Creatine Kinase B (CKB)* is a key enzyme in cellular energy balance and has emerged as a promising biomarker in several cancers such as OC [79, 80, 81]. Its suppression slows tumor growth, enhances chemotherapy response, and disrupts metabolic pathways [16]. With a 22‐ to 36‐fold increase in cancer tissues, CKB shows strong potential as a non‐invasive early detection marker [80].

*Bikunin* is a heavily glycosylated protein and a Kunitz‐type protease inhibitor [82]. Research shows that it suppresses urokinase‐type plasminogen activator (uPA) and its receptor (uPAR), which are involved in cancer invasion and metastasis [83, 84]. In ovarian cancer cells, bikunin gene transfection reduced uPA/uPAR expression and decreased invasiveness [85].

*Osteopontin (OPN)* is a multifaceted secreted extracellular matrix glycoprotein involved in wound healing, inflammation, immune responses, and cancer progression [86]. It is often found at high levels in many cancers. Elevated serum OPN is used to help diagnose and predict outcomes in cancers like liver and breast cancer [87, 88]. In ovarian cancer, OPN levels are usually higher, making it a promising diagnostic marker [89]. Recent research shows that while both OPN and CA125 rise in ovarian cancer, OPN is better at distinguishing malignant tumors from benign ones, suggesting it could be a useful complement to CA125 for more accurate diagnosis [90].

## 3 Fundamentals of Machine Learning in Healthcare

Machine learning (ML) is a powerful tool that identifies patterns in data and associates them with specific categories to build predictive models [91, 92]. The process involves selecting, exploring, and cleaning data, followed by analysis with various algorithms. Outcomes can be binary, such as predicting breast cancer recurrence based on factors like tumor size and age, and models are evaluated using metrics like AUC, precision, and sensitivity. Typically, models are trained on 70% of the data and tested on the remaining 30% to ensure effectiveness [93]. ML algorithms are categorized into supervised, unsupervised, and reinforcement learning. Supervised learning uses labeled data for classification (e.g., patient survival) or regression (e.g., healthcare costs) [94, 95]. Unsupervised learning uncovers patterns in unlabeled data through clustering (e.g., k‐means) or association rules (e.g., market basket analysis) [96, 97]. Reinforcement learning, driven by rewards, optimizes actions in applications like treatment planning [98].

In healthcare, particularly oncology, ML revolutionizes cancer prediction, diagnosis, and treatment [99, 100]. It enhances early detection by analyzing medical images, genomic data, and electronic health records [101]. ML predicts cancer risk using genetic and lifestyle factors [102, 103], forecasts disease progression and survival [104, 105], and predicts treatment responses and recurrence [106, 107, 108, 109]. It also automates tumor classification [110, 111], supports drug discovery [112, 113], predicts immunotherapy responses [114, 115], and optimizes radiation therapy [116, 117]. OC, often diagnosed late due to subtle symptoms and inadequate screening, presents significant challenges. ML, leveraging molecular biomarkers, offers potential for improving early detection and risk stratification, with further advancements explored in subsequent sections [118].

## 4 Review Methodology

This narrative review employed a structured literature search and evaluation approach to identify and analyze relevant studies that applied ML techniques to biomarker data for predicting diagnosis, prognosis, or treatment response in OC. The methodology comprised a targeted search strategy, application of eligibility criteria, selection and review of studies, data extraction, quality assessment, and data synthesis.

### 4.1 Search Strategy

A targeted literature search was conducted in PubMed/MEDLINE, Scopus, and Web of Science to identify peer‐reviewed articles published between January 2000 and March 2025. The search strategy focused on identifying original research articles that applied ML algorithms to serum or plasma biomarker data for the prediction or management of ovarian cancer. Search terms included combinations of the following keywords and Medical Subject Headings (MeSH): *“ovarian cancer”*, *“machine learning”*, *“biomarkers”*, *“predictive model”*, *“early diagnosis”*, *“treatment response”*, as well as names of specific ML algorithms such as “support vector machine”, *“random forest”*, *“neural network”*, and *“deep learning”*. Boolean operators (“AND”, “OR”) were used to enhance retrieval sensitivity. Searches were limited to English‐language articles published in peer‐reviewed journals. To improve completeness, reference lists of relevant articles and recent reviews were manually screened for additional studies.

### 4.2 Inclusion and Exclusion Criteria

Studies were included if they met the following criteria:

- Applied supervised or unsupervised ML algorithms;
- Utilized serum or plasma biomarkers as input features;
- Focused on ovarian cancer diagnosis, prognosis, or treatment response prediction;
- Reported sufficient methodological detail regarding the ML approach, validation strategy, and performance metrics (e.g., AUC, accuracy, sensitivity, specificity);
- Published as original articles in peer‐reviewed journals and written in English.

Exclusion criteria were:

- Use of imaging data only, without biomarker or clinical inputs;
- Focus on cancer types other than ovarian cancer;
- Review articles, editorials, letters, or conference abstracts lacking complete methodological information.
- Publications in languages other than English.

### 4.3 Study Selection

All titles and abstracts retrieved from the search were independently screened by two reviewers. Full‐text articles were then assessed for eligibility based on the predefined inclusion and exclusion criteria. Disagreements were resolved through discussion, or by involving a third reviewer if needed. A total of 17 studies met the eligibility criteria and were included in the final analysis.

### 4.4 Data Extraction

Data were extracted from the included studies using a standardized data extraction form. Extracted information included:

- Study design and population characteristics (e.g., sample size, age, case/control distribution);
- Type of data used (e.g., cross‐sectional, longitudinal);
- Clinical task addressed (e.g., early detection, diagnostic classification, prognosis, treatment response);
- Biomarkers used (e.g., CA‐125, HE4);
- Machine learning algorithms applied (e.g., support vector machines, random forests, deep learning methods);
- Feature selection methods and model validation strategies;
- Model performance metrics (e.g., AUC, accuracy, sensitivity, specificity).

All data were independently verified by two reviewers to ensure consistency and accuracy.

### 4.5 Quality Assessment

The methodological quality of the included studies was evaluated based on the representativeness and size of the study population, the appropriateness and transparency of the machine learning methodology (including algorithm selection and validation strategies), and the completeness of performance metric reporting. Studies that used suitable validation methods—such as cross‐validation or external testing—and provided clear performance outcomes were considered methodologically robust.

### 4.6 Data Synthesis

Due to the heterogeneity in biomarkers, ML algorithms, and study designs, a narrative synthesis was conducted. Studies were categorized into three application domains:

- Diagnostic models for early detection of ovarian cancer;
- Prognostic models for survival prediction;
- Treatment response models.

Where possible, model performance metrics were compared across studies, and emerging trends were summarized in Tables 2, 4.

**TABLE 2:** Summary of investigations on biomarker‐driven machine learning for ovarian cancer prediction.

Study	Data type	Clinical task	Patient data	Age	Top features	Reported AUC/Accuracy
M. Lu et al. [119]	Cross‐sectional	Diagnostic classification	OC = 171 BOT = 178	OC = 53, BOT = 36	HE4, CEA	**DT**: Accuracy: 95.6% and AUC: 0.949 **ROMA**: Accuracy: 92.1% and AUC: 0.943 **LR**: Accuracy: 97.4% and AUC: 0.969
Laios A et al. [120]	Cross‐sectional	Prognosis	571 EOC	63.5 + 11.2	Intraoperative Mapping of Ovarian Cancer Score, Surgical Complexity Score, Peritoneal Carcinomatosis Index, Tumor Bulk Size, Age	**XGBoost:** AUC**: 0.866**
Ma et al. [121]	Cross‐sectional	Diagnostic classification	156 EOC	57.89 ± 9.01	Mesenchyma‐CTC Percentage, Total CTC Count, CA‐125, CRP, Fibrinogen, Albumin	**RF**: AUC, 0.796 **GBM**: AUC, 0.748 **LR**: AUC,0.692 **NN**: AUC, 0.730 **SVM**: AUC, 0.726
Laios et al. [122]	Cross‐sectional	Prognosis	560 EOC	64 ± 11	Peritoneal Carcinomatosis Index, Intraoperative Mapping of Ovarian Cancer, Surgical Complexity Score, Pre‐surgery CA‐125, Surgeon's Age, Case Volume, Years of Experience	**XGBoost**: AUC: 0.77 **DNN**: AUC: 0.739
Kawakami et al. [123]	Cross‐sectional	Diagnostic classification	334 EOC and 101 BOT	52.2 (19–87)	Age, CA‐125, Albumin, Lactate Dehydrogenase, Lymphocyte Count, Sodium, Fibrinogen, CRP	**LR**: Accuracy: 86.7% and AUC: 0.897 **GBM**: Accuracy: 93.7% and AUC: 0.976 **SVM**: Accuracy: 90.5% and AUC: 0.939 **RF**: Accuracy: 92.4% and AUC: 0.968 **CRF**: Accuracy: 93.7% and AUC: 0.978 **NB**: Accuracy: 88.6% and AUC: 0.954 **NN**: Accuracy: 88.0% and AUC: 0.883 **EN**: Accuracy: 91.8% and AUC: 0.966
Enshaei et al. [124]	Cross‐sectional	Prognosis	668 EOC		Age, FIGO stage, grade, histologic subtype, preoperative CA‐125 levels, surgical outcome	**ANN**: Accuracy: 93% and AUC: 0.74 **DT**: Limited Predictive Capacity **NB**: Lower than ANN **LR**: Lower than ANN and AUC: 0.62
Laios et al. [125]	Cross‐sectional	Treatment response	154 OC	64.4 + 10.5	Age, BMI, Charlson Comorbidity Index Surgical Complexity Score, Diseases Score, Pre‐treatment CA‐125.	**KNN**: Accuracy: 65.8% **LR**: Accuracy: 63.4%
Laios et al. [126]	Cross‐sectional	Prognosis	209 HGSOC	64.6 ± 10.6 (41–85)	Standard chemotherapy, Disease Score, Surgical Complexity Score, Residual Disease, Performance Status, Primary Debulking Surgery	**SVM**: Accuracy: 72.9%, AUC: 0.66 **KNN:** Accuracy: 71.8%, AUC: 0.62 **LR**: Accuracy: 66.5%, AUC: 0.59 **NB:** Accuracy: 66.0%, AUC: 0.63
Feng et al. [127]	Cross‐sectional	Prognosis	98 OC	57	Age, Monocyte‐to‐Lymphocyte Ratio, diferentiation status, CA125 level, NE, ascites cytology, Lymphocyte Percentage	**DT**, AUC:0.69 **LR**, AUC: 0.55
Ledger et al. [128]	Cross‐sectional	Diagnostic classification	5909 OC	49 (36–62)	Age, CA125, proportion of solid tissue, maximum diameter of the lesion, presence of shadows, presence of ascites, number of papillary projections	**Ridge MLR**: AUC: 0.90 **SVM**, AUC: 0.89 **MLR**, AUC: 0.92 **RF**, AUC: 0.92 **XGBoost**, AUC: 0.92 **NN**, AUC: 0.92
Kucukakcali Z et al. [129]	Cross‐sectional	Diagnostic classification	171 MOC and 178 BOT	45.05 ± 15.13	CA72‐4, HE4, Lymphocyte Percentage, Albumin, Eosinophil Percentage, Blood Urea Nitrogen, Red Blood Cells, Neutrophils, Mean Corpuscular Volume	**XGBoost:** Accuracy: 89.5% **SGBoost:** Accuracy 84.2%:
Sun et al. [130]	Cross‐sectional	Diagnostic classification	BOT:304 OC:311 Borderline OC: 98	34–48	Age, BMI, CA125, CEA, CA199, CA72‐4, HE4	**RF**: AUC: 0.86, Accuracy: 99.82% **LR**: AUC: 0.95, Accuracy: 78.0% **SVM**: AUC: 0.85, Accuracy: 69.6% **KNN**: AUC: 0.90, Accuracy: 74.8%
Abrego et al. [131]	Longitudinal	Early detection	HI: 180 OC: 44	[50.3, 78.8] [52.0, 77.4]	CA125, HE4, Glycodelin	**BCP** (CA125 + HE4): AUC: 0.971, Sensitivity: 96.7% **BCP** (CA125 only): AUC: 0.949, Sensitivity: 90.89% **RNN** (CA125 + HE4): AUC: 0.987 Sensitivity: 96.7% **RNN** (CA125 only): AUC: 0.953, Sensitivity: 92.1%
Lavanya et al. [132]	Cross‐sectional	Diagnostic classification	HI: 171 OC: 176	[23–68]	CA‐125, Indirect Bilirubin, Hemoglobin, Ca, CA19‐9, Platelet Count, Lymphocyte Percentage, Procalcitonin	**SVM**: Accuracy: 85% **SELM**: Accuracy: 89%
Feng et al. [133]	Cross‐sectional	Diagnostic classification	OC: 185 Control group: (MOT: 138, BOT: 339, HI: 92)	OC: [16–83] Control group: : [20–85].	CA125, CA15‐3, CA72‐4, Estradiol, Progesterone, Blood glucose, lipid levels (TG, LDL‐C/HDL‐C), CRP, Neutrophil‐to‐Lymphocyte Ratio, Red Cell Distribution Width	**BP‐NN**: AUC: 0.948, Sensitivity: 91.9%, Specificity: 86.9%
Ayyoubzadeh et al. [134]	Cross‐sectional	Diagnostic classification	OC: 178 BOC: 171	[15–83] Mean: 45 (±15.1)	HE4, CA‐125, Neutrophil Ratio	**RF**: Accuracy: 86.75%, AUC: 0.925 **SVM**: Accuracy: 85.25%, AUC: 0.910 **DT**: Accuracy: 82.91%, AUC: 0.799 **ANN**: Accuracy: 79.35%, AUC: 0.890
Li et al. [135]	Cross‐sectional	Diagnostic classification	BOC: 534 OC: 224	41.9 ± 16.1	Tumor size (MR/CT), HE4, CA‐125, platelet count, lymphocyte ratio	**DT:** AUC = 0.86

**Abbreviations:** ANN, artificial neural network; BCP, Bayesian change‐point; BN, Bayesian network; BOT, benign ovarian tumors; BP‐NN, backpropagation neural network; CRF, conditional random forest; CRF, conditional RF; DNN, deep neural network; DT, decision tree; EOC, epithelial ovarian cancer; GBM, gradient boosting machine; KNN, K‐nearest neighbor; LR, logistic regression; MLR, multinomial logistic regression; MOT, malignant otolaryngology tumor; NB, naive bayes; NN, neural network; OC, ovarian cancer; RF, random forest; RNN, recurrent neural networks; SELM, stacked ensemble learning model; SGB, stochastic gradient boosting; SVM, support vector machine; XGBoost, extreme gradient boosting.

## 5 Review Findings: Machine Learning Applications in Ovarian Cancer

ML is increasingly transforming ovarian cancer research by enabling data‐driven models for non‐invasive prediction, diagnosis, prognosis, and treatment planning. In this section, we categorize and summarize key studies based on four core clinical objectives:

- *Early detection*—identifying preclinical or asymptomatic ovarian cancer, particularly in high‐risk populations;
- *Diagnostic classification*—distinguishing between benign and malignant ovarian tumors;
- *Prognosis*—predicting outcomes such as progression‐free survival, overall survival, or recurrence risk;
- *Treatment response*—forecasting therapeutic outcomes, including chemotherapy efficacy or optimal cytoreductive surgery success.

We reviewed and analyzed 17 studies that used ML techniques in these clinical domains, focusing on the algorithms applied, biomarker inputs, dataset sizes, and performance metrics. Table 2 presents a consolidated summary of these investigations, offering a practical overview of how ML is currently being leveraged in ovarian cancer care. In summary, across all studies reviewed, ensemble‐based ML models such as Random Forest and XGBoost consistently outperformed other algorithms in terms of predictive accuracy, particularly when applied to large, multi‐feature datasets. Biomarkers like CA‐125 and HE4 remained core components in most models, though their diagnostic utility significantly improved when combined with inflammatory, metabolic, and radiomic features. The clinical applicability of these models was further enhanced through the use of balanced datasets, robust cross‐validation strategies, and multi‐modal data integration. These findings underscore the growing potential of ML‐driven approaches for personalized, non‐invasive ovarian cancer prediction, especially when applied across diverse clinical tasks such as early detection, diagnosis, prognosis, and treatment response.

### 5.1 Categorization of Biomarkers in Ovarian Cancer Prediction

The biomarkers utilized in OC prediction can be classified into four main categories: tumor markers, inflammatory markers, hormonal and metabolic markers, and hematologic parameters. Each of these categories plays a distinct role in detecting, diagnosing, and prognosticating ovarian cancer. By analyzing these biomarkers using machine learning models, researchers have improved predictive accuracy and clinical decision‐making in distinguishing between ovarian cancer, benign ovarian tumors, and healthy individuals. The studies analyzed in this review demonstrate the impact of different biomarker categories and how machine learning techniques enhance ovarian cancer detection and prognosis. Table 3 summarizes the categorization and significance of biomarkers in ML‐based OC prediction.

**TABLE 3:** Categorization and significance of biomarkers in machine learning‐based ovarian cancer prediction.

Feature category	Feature	Biological role	Impact on prediction
Tumor markers	CA‐125	Most widely used ovarian cancer biomarker; elevated in > 80% of advanced cases but lacks specificity in early stages	Strong predictor but insufficient alone due to false positives in benign conditions
	HE4	Complements CA‐125; more specific for ovarian cancer and useful for early‐stage detection	Increased specificity when combined with CA‐125 (ROMA algorithm)
	CEA .	Associated with mucinous ovarian tumors and gastrointestinal cancers	Moderate predictor, more useful when combined with CA‐125
	CA19‐9	Originally a pancreatic cancer marker, but elevated in ovarian cancer	Useful in differentiating benign vs. malignant tumors
	CA72‐4	Associated with ovarian and gastric cancers; complements CA‐125 for detection	Improves early‐stage detection; enhances CA‐125 specificity
Inflammatory markers	Neutrophil‐to‐lymphocyte ratio (NLR)	Indicator of chronic inflammation; high NLR is linked to poor prognosis in ovarian cancer	Strong prognostic value; included in multiple predictive models
	CRP	Acute phase protein indicating systemic inflammation; elevated in advanced ovarian cancer	Predicts tumor aggressiveness and treatment response
	LDH	Metabolic enzyme elevated in rapidly growing tumors; associated with tumor burden	Linked to tumor stage and recurrence risk
	Lymphocyte percentage (LYM%)	Measures immune response; lower values indicate immune suppression by tumors	Useful for prognosis and response prediction
Hormonal and metabolic markers	Estradiol (E2)	Estrogen hormone involved in ovarian cancer proliferation	Helps differentiate hormone‐dependent tumors
	Progesterone (P4)	Opposes estrogen effects; lower levels linked to cancer progression	May help classify hormone‐sensitive subtypes
	Blood glucose (FPG)	Metabolic dysregulation in cancer; ovarian tumors alter glucose metabolism	Linked to insulin resistance and tumor growth
	Lipid levels (TG, LDL‐C/HDL‐C)	Lipid metabolism is disrupted in cancer progression	May predict tumor aggressiveness
Hematologic parameters	Hemoglobin (HGB)	Lower levels indicate cancer‐associated anemia	Associated with disease progression
	Platelet count (PLT)	Tumor‐induced platelet activation promotes angiogenesis and metastasis	Predicts tumor progression and recurrence
	Red cell distribution width (RDW)	Marker of inflammation and anemia; linked to tumor burden	Useful for prognosis

Biomarkers such as CA‐125 and HE4 are critical for OC prediction and can be measured either as single‐time‐point (cross‐sectional) values or as serial measurements over time (longitudinal data). In contrast, cross‐sectional data are more commonly used in routine diagnostics due to their simplicity and availability. The choice of data type influences the suitability of ML algorithms, with models like RNNs designed to leverage longitudinal data, while conventional ML methods (e.g., Random Forest, SVM) typically use cross‐sectional features. This distinction is critical when comparing model performance across studies.

#### 5.1.1 Tumor Markers: Key Indicators of Malignancy

Tumor markers are widely used in OC prediction because they are directly associated with tumor growth, progression, and malignancy. Among these, CA‐125 is the most frequently analyzed biomarker and remains a gold standard for OC detection. It is a glycoprotein expressed in the epithelium of ovarian tumors, and its levels rise in response to peritoneal inflammation and cancer progression. Nearly all studies incorporated CA‐125, with results demonstrating its high predictive power. In a study with 5909 patients, machine learning models using CA‐125 achieved an AUC of approximately 0.92, demonstrating its effectiveness in cancer classification [128]. Other models, such as RF, RNN, and XGBoost, also achieved high accuracy when incorporating CA‐125, reinforcing its diagnostic value [121, 123, 130, 131, 132, 134].

Beyond CA‐125, HE4 has emerged as a powerful complementary biomarker. Unlike CA‐125, which can be elevated in benign conditions, HE4 has demonstrated higher specificity for OC. Several studies showed that combining HE4 with CA‐125 significantly improved prediction models, with reported AUC values exceeding 0.90 [129, 131, 134]. A study analyzing 178 OC and 171 BOT cases found that a model using HE4 and CA‐125 in a RF approach achieved an AUC of 0.925 [134]. The combination of HE4 and CA‐125 is particularly useful in differentiating ovarian cancer from benign ovarian tumors, making it an important feature in predictive modeling. A recent study utilizing a decision tree‐based model demonstrated that integrating ROMA index, HE4, CA‐125, platelet count, and lymphocyte ratio significantly enhanced malignancy prediction, achieving an AUC of 0.86 [135]. The model identified ROMA_after as the most informative feature, forming the root node of the decision tree. Furthermore, the study confirmed that features such as tumor size (MR/CT), platelet count, and lymphocyte ratio played a crucial role in classifying ovarian tumors.

Additional tumor markers such as CEA and CA72‐4 were evaluated in multiple studies. CEA is commonly associated with gastrointestinal malignancies but has shown utility in ovarian cancer prediction, particularly when combined with other markers. A study incorporating CA72‐4, along with other tumor markers, found that it contributed to a Random Forest model with an AUC of 0.86 [129, 130]. Similarly, Glycodelin, a glycoprotein involved in immune modulation and cancer cell survival, was examined in a study where the combination of Glycodelin, CA‐125, and HE4 led to an improved AUC of 0.987 in an RNN model [131]. These findings suggest that tumor markers play a critical role in ovarian cancer prediction and that their integration in machine learning models can significantly improve classification performance. The UKCTOCS demonstrated the use of longitudinal CA‐125 measurements for early detection, achieving a 24.5% reduction in stage IV disease but no significant mortality benefit [22]. This suggests that machine learning models, particularly those leveraging longitudinal data like Abrego et al. [131], could improve early detection by capturing temporal biomarker trends, potentially addressing the limitations of traditional screening approaches.

#### 5.1.2 Inflammatory Markers: Linking Systemic Inflammation to Cancer Progression

Chronic inflammation is known to contribute to tumorigenesis, making inflammatory biomarkers essential for OC prediction. Inflammatory processes can lead to DNA damage, promote angiogenesis, and facilitate tumor cell survival and metastasis. One of the most commonly analyzed inflammatory markers is CRP, which was included in multiple studies. Elevated CRP levels are often observed in cancer patients due to tumor‐induced inflammation and immune response dysregulation. In one study evaluating 156 patients, CRP contributed to models that achieved AUC values above 0.79 [121, 123, 133].

Another key inflammatory marker is Fibrinogen, which is often elevated in patients with malignancies. Fibrinogen plays a role in blood clot formation, but in cancer, it has been linked to tumor cell adhesion, metastasis, and immune evasion. In the same study, Fibrinogen was included alongside CRP in machine learning models, further improving predictive accuracy [121, 123]. Similarly, Lactate Dehydrogenase (LDH), an enzyme involved in glycolysis, was examined in an analysis of 334 EOC and 101 BOT patients. Elevated LDH levels are associated with tumor metabolism, hypoxia, and aggressive tumor behavior, contributing to a GBM model that reached an AUC of 0.976 [123].

Another important inflammatory parameter is the Neutrophil‐to‐Lymphocyte Ratio (NLR), which reflects immune system dysregulation in cancer patients. High NLR is associated with increased tumor‐associated inflammation and poor prognosis. Machine learning models incorporating NLR demonstrated improved sensitivity and specificity [133, 134]. Also, a recent study showed that the lymphocyte ratio significantly contributed to malignancy prediction [135]. These findings highlight the importance of inflammatory biomarkers in machine learning‐based ovarian cancer prediction.

#### 5.1.3 Hormonal and Metabolic Markers: Indicators of Cancer‐Related Dysregulation

Hormonal and metabolic dysregulation are key features of ovarian cancer, making these biomarkers valuable for predictive modeling. Estradiol and Progesterone, the two primary ovarian hormones, are implicated in ovarian cancer progression through estrogen receptor signaling. Their predictive value was demonstrated in machine learning models that showed improved classification performance [133]. Metabolic alterations, including changes in blood glucose levels and lipid profiles (TG, LDL‐C/HDL‐C ratios), are also linked to cancer development. These markers were highlighted as significant predictors of ovarian cancer, contributing to improved model accuracy [133]. Additionally, Albumin, an important nutritional marker, was found to be associated with ovarian cancer progression [123, 129].

Cancer cells exhibit altered metabolism, known as the Warburg effect, where they preferentially use glycolysis for energy production even in the presence of oxygen [136]. Blood glucose levels and lipid profiles, such as triglycerides (TG) and LDL‐C/HDL‐C ratios, were evaluated as potential indicators of metabolic changes associated with OC. Altered lipid metabolism contributes to tumor growth, inflammation, and drug resistance [137]. One study demonstrated that integrating these metabolic markers into predictive models improved performance by capturing cancer‐related metabolic dysregulations [133]. These findings suggest that hormonal and metabolic markers play a crucial role in ovarian cancer detection and should be considered in future predictive modeling efforts.

The predictive power of metabolic markers was further enhanced by machine learning techniques such as DNN, XGBoost, and SVM. These models showed improved classification accuracy when incorporating metabolic parameters alongside tumor and inflammatory markers, emphasizing the need for a multi‐biomarker approach in ovarian cancer prediction.

#### 5.1.4 Hematologic Parameters: Reflecting Systemic Changes in Malignancy

Hematologic parameters provide valuable insights into systemic changes associated with ovarian cancer. Lymphocyte percentage (LYM %), a key indicator of immune function, was identified as an important predictor, contributing to high‐performance classification models [123, 129, 132]. Other hematologic markers, such as Platelet Count (PLT) and Mean Corpuscular Volume (MCV), were also investigated, further supporting their role in ovarian cancer detection [132, 135]. Platelets play an essential role in tumor metastasis through tumor‐induced platelet aggregation, which protects cancer cells from immune attacks [138]. These findings emphasize that changes in blood cell composition, often linked to cancer‐related inflammatory responses, can enhance ovarian cancer prediction. Red cell distribution width (RDW), a measure of erythrocyte size variation, was linked to tumor‐induced anemia and inflammation, improving the sensitivity of machine learning models [133]. RDW is often elevated in cancer patients due to bone marrow stress, oxidative damage, and anemia, which are common in malignancies [139]. These markers were successfully integrated into machine learning models, including NN, SVM, and XBoost, with reported accuracy levels exceeding 85% in multiple studies [132, 133]. The inclusion of hematologic parameters alongside tumor, inflammatory, and metabolic markers further enhances ovarian cancer prediction models.

The integration of tumor markers, inflammatory markers, hormonal/metabolic markers, and hematologic parameters significantly improves the accuracy and robustness of ovarian cancer prediction models. Among machine learning techniques, RF, XGBoost, and ANNs consistently outperformed others, achieving AUC values above 0.85. In large‐scale studies, models incorporating tumor markers, inflammatory markers, and machine learning approaches achieved an AUC of 0.92 [128]. Similarly, studies evaluating smaller datasets found that integrating multiple biomarker categories led to improved classification accuracy.

### 5.2 Performance of Machine Learning Models in Ovarian Cancer Prediction

The performance of ML models in OC prediction is influenced not only by the algorithm but also by the type of data used. Longitudinal data, which include serial measurements of biomarkers or clinical parameters over time, capture temporal dynamics and are particularly suited for models like RNNs, which are designed to model sequential patterns [140]. In contrast, cross‐sectional data, which consist of single‐time‐point measurements, are commonly used in conventional ML models such as RF, XGBoost, SVM, and LR. Among the studies reviewed, Abrego et al. [131] utilized longitudinal data for RNN models, leveraging serial CA‐125 and HE4 measurements to achieve high diagnostic accuracy (AUC = 0.987). Other studies on cross‐sectional data incorporated single‐time‐point biomarker levels and clinical features. This distinction in data types complicates direct comparisons between RNNs and conventional ML methods, as longitudinal data may provide more predictive information due to temporal trends. To ensure fair comparisons, this review separates discussions of model performance by data type where possible and highlights the influence of data characteristics on reported outcomes.

#### 5.2.1 Machine Learning in Ovarian Cancer Diagnosis

One of the most important applications of ML in ovarian cancer research is distinguishing malignant OC from BOT and healthy individuals. Multiple studies in this review applied supervised learning models to classify OC using biomarker panels, inflammatory markers, and imaging features. The most commonly used algorithms included RF, XGBoost, and NN, which consistently outperformed traditional statistical approaches. Several studies demonstrated that ensemble methods, particularly RF and XGBoost, achieved the highest classification accuracy. For models using cross‐sectional data, one study analyzing 171 OC and 178 BOT cases reported an XGBoost accuracy of 89.5% with biomarkers like CA‐125, HE4, and hematologic parameters [129]. In contrast, Abrego et al. [131] utilized longitudinal data with serial CA‐125 and HE4 measurements to detect ovarian cancer in healthy individuals (HI:180, OC:44), making it suitable for early detection. The high AUC (0.987) with an RNN model highlights its focus on capturing temporal patterns for early identification. The high performance of the RNN model may be partly attributed to the temporal patterns captured by longitudinal data, which provide additional predictive information compared to the single‐time point data used in most conventional ML models (e.g., RF, SVM, LR). Similarly, a study analyzing Borderline OC, BOT, and malignant OC cases (304, 311, and 98 patients, respectively) reported an RF accuracy of 99.82% and an AUC of 0.86 [130].

DT and LR models also performed well in certain studies. A study analyzing 171 OC and 178 BOT cases reported that a DT model achieved 95.6% accuracy and an AUC of 0.949, while LR reached an AUC of 0.969 [119]. These results suggest that traditional ML approaches can still be effective, particularly when biomarkers such as HE4 and CEA are included. However, in another study evaluating 156 EOC cases, LR performed poorly (AUC = 0.692) compared to ensemble methods, highlighting variability across different datasets [121]. Similarly, a DT model applied to a 98‐patient dataset achieved an AUC of 0.69, confirming that tree‐based methods struggle with complex biomarker interactions [127]. Furthermore, in a larger dataset comprising 224 benign ovarian tumors and 534 malignant tumors, a classification and regression tree (CART) model achieved an AUC of 0.86 for distinguishing between benign and malignant ovarian cancer. This model using only imaging indicators or biomarkers was outperformed by a decision tree model that also used clinical indicators and preoperative circulating blood cells. These findings suggest that integrating multiple clinical and hematologic features enhances the accuracy of ovarian cancer prediction, leading to more precise differentiation between benign and malignant cases [135].

SVM also demonstrated strong classification performance, particularly when combined with tumor biomarkers. A study analyzing 178 OC and 171 BOT cases found that SVM achieved an AUC of 0.910 [134]. Another study evaluating 334 EOC and 101 BOT cases showed that SVM achieved an accuracy of 90.5% and an AUC of 0.939, outperforming traditional LR models [123]. These findings suggest that ensemble learning (RF, XGBoost) and deep learning models (RNN, ANN) provide the highest accuracy for ovarian cancer diagnosis, while traditional methods like LR and DT can still be valuable in specific biomarker‐based models.

#### 5.2.2 Machine Learning in Survival Prediction and Prognosis

ML models have also been applied to predict overall survival and progression‐free survival by integrating clinical and biomarker data. The most effective models for survival prediction were XGBoost and DNNs. A study analyzing 571 ovarian cancer patients applied an XGBoost model incorporating intraoperative features and tumor burden scores, achieving an AUC of 0.866 [120]. Another study evaluating 560 EOC patients found that XGBoost models integrating Peritoneal Carcinomatosis Index and Surgical Complexity Scores achieved an AUC of 0.77, making them effective for survival estimation [122].

Other studies explored different ML classifiers for survival prediction. In a dataset of 209 high‐grade serous ovarian cancer cases, SVM achieved an accuracy of 72.9% (AUC = 0.66), while KNN and NB models performed slightly worse [126]. Additionally, another study found that preoperative leukocyte levels (MO/LY ratio) were predictive of survival outcomes, with the DT model (AUC:0.69) demonstrating higher predictive accuracy than traditional clinical assessments [127]. A DNN model incorporating surgical complexity, tumor bulk size, and patient age reached an AUC of 0.739, indicating its potential in survival modeling [122]. Conversely, LR and NB showed lower predictive ability. In a study of 668 EOC cases, LR achieved an AUC of only 0.62, while Naïve Bayes performed even worse [124]. These results confirm that XGBoost and deep learning models (DNN, ANN) are the best choices for survival prediction.

#### 5.2.3 Machine Learning in Treatment Response Prediction

Predicting treatment response, including cytoreduction feasibility and chemotherapy effectiveness, is essential for improving ovarian cancer management. Several studies have applied machine learning models to assess treatment outcomes using clinical parameters, surgical complexity scores, tumor burden indices, and biomarker levels. Among ML models, XGBoost and DNNs have demonstrated strong predictive power in treatment response evaluation. One study analyzing 571 ovarian cancer patients used an XGBoost model incorporating intraoperative tumor burden scores, achieving an AUC of 0.866 [120]. This model effectively predicted surgical outcomes, indicating that intraoperative features play a crucial role in assessing cytoreduction feasibility. Another study, which included 560 EOC patients, applied XGBoost and DNN models to predict treatment response based on peritoneal carcinomatosis index, surgical complexity score, and preoperative CA‐125 levels. The XGBoost model achieved an AUC of 0.77, while the DNN model reached an AUC of 0.739, demonstrating the potential of ML models in guiding surgical decision‐making [122].

In a separate study analyzing 154 ovarian cancer cases, machine learning models incorporating age, BMI, Charlson Comorbidity Index, pre‐treatment CA‐125, and surgical complexity scores were used to predict treatment response. A KNN model achieved an accuracy of 65.8%, while LR performed slightly worse with an accuracy of 63.4% [125]. These results suggest that traditional models like LR and KNN may be less effective than ensemble methods and deep learning approaches for predicting treatment feasibility. Overall, these findings confirm that XGBoost and DNNs outperform traditional statistical approaches like LR and KNN in treatment response prediction. The integration of surgical complexity scores, tumor burden indices, and preoperative biomarkers into ML models enhances their ability to guide clinical decision‐making and improve treatment planning.

The effectiveness of ML models in OC prediction varies by task and data type. For diagnosis, RNNs achieved the highest performance (AUC = 0.987) using longitudinal CA‐125 and HE4 measurements [131], likely benefiting from temporal patterns in the data. In contrast, RF and XGBoost models, which used cross‐sectional biomarker and clinical data, achieved high accuracy (e.g., 99.82% for RF [130]) but may not capture temporal dynamics. For survival prediction, XGBoost models using cross‐sectional intraoperative features excelled (AUC = 0.866 [120]). These findings underscore the importance of considering data type when comparing model performance, as longitudinal data may enhance predictive accuracy for certain algorithms like RNNs. For treatment response prediction, RF demonstrated the highest accuracy of 99.82%, outperforming other models. A summary of the best‐performing ML models for each task, including their highest reported performance and key predictive features, is presented in Table 4 below.

**TABLE 4:** Best‐performing ML models for ovarian cancer prediction.

Prediction task	Best models	Highest performance	Key features used	Data type
Ovarian cancer diagnosis	RF, XGBoost, RNN	**AUC = 0.987** (RNN with CA‐125 and HE4)	CA‐125, HE4, CEA, CA72‐4, clinical parameters	Longitudinal (RNN), Cross‐sectional (RF, XGBoost)
Survival prediction	XGBoost, DNN	**AUC = 0.866** (XGBoost with intraoperative features)	Tumor burden indices, PCI, SCS, CA‐125	Cross‐sectional
Treatment response	RF, SVM, KNN	**Accuracy = 99.82%** (RF with CA‐125 and surgical scores)	CA‐125, surgical complexity, comorbidity indices	Cross‐sectional

### 5.3 Advantages of Machine Learning–Based Biomarker Studies

ML offers transformative potential in OC research by enhancing diagnostic accuracy, integrating multiple biomarkers, improving model interpretability, fusing multi‐modal data, and supporting clinical decision‐making. ML models achieve high classification accuracy in distinguishing OC from benign conditions, with random forest (RF) models reaching up to 99.82% accuracy and an AUC of 0.925 [130, 134]. Ensemble methods like RF and gradient boosting machines (GBM) consistently outperform traditional models, with AUCs above 0.96 [123]. Deep learning, such as artificial neural networks (ANNs), achieves 93% accuracy in survival prediction, surpassing logistic regression [124].

Combining biomarkers like CA‐125, HE4, and CA72‐4 enhances sensitivity and specificity, with a Bayesian Change‐Point Model achieving an AUC of 0.971 [131] and XGBoost reaching 89.5% accuracy [129]. Incorporating clinical factors (e.g., age, FIGO stage) further improves survival prediction [124]. Explainable AI techniques, such as Shapley Additive Explanations (SHAP) analysis, enhance model transparency by identifying key predictors like tumor burden indicators and biomarkers, making ML clinically actionable [120, 122, 132]. Multi‐modal data fusion, integrating radiomics, genomics, and biomarkers, significantly boosts accuracy. ML also excels in prognosis, with ANNs and SVMs predicting survival outcomes at 73 to 93% accuracy and in treatment planning. Also, ML plays a key role in clinical decision‐making, helping oncologists optimize treatment plans. One used XGBoost with SHAP to predict the complexity of cytoreductive surgery, enabling surgeons to make informed preoperative decisions [122]. By leveraging multi‐modal data, interpretable AI, and deep learning, ML revolutionizes OC early detection, risk stratification, and personalized treatment, with the potential to improve patient outcomes upon clinical validation.

### 5.4 Limitations of Machine Learning–Based Biomarker Studies

Despite the promise of ML in OC prediction, several limitations hinder generalizability and clinical applicability. Small dataset sizes, often as low as 44 to 209 cases, reduce statistical power and model robustness, particularly in single‐center studies [126, 131]. Lack of external validation datasets leads to overfitting, with models showing high training accuracy (e.g., near‐perfect) but significant performance drops (e.g., 21% or down to 78.9%) on external data [130, 133]. The UKCTOCS study highlights the limited sensitivity of CA‐125 for early detection [22], a challenge compounded by many studies excluding imaging (e.g., CT/MRI radiomics) and genomic data (e.g., BRCA mutations), which could enhance model accuracy [121, 122]. Classifying borderline tumors and early‐stage cancers is challenging due to subjective histopathological interpretations, low biomarker specificity (e.g., CA‐125, HE4), and suboptimal screening methods [128, 130, 132]. Overfitting is prevalent in complex models trained on small or imbalanced datasets, necessitating cross‐validation, regularization, and diverse data [126, 129, 141, 142]. Short follow‐up periods limit survival and treatment response predictions, requiring longitudinal data collection [121, 127]. Addressing these limitations through larger, multi‐center datasets, multi‐modal data integration, and robust validation is critical for reliable ML‐driven OC diagnostic tools.

## 6 Future Directions

As ML continues to evolve in the field of ovarian cancer prediction, addressing current limitations is crucial for real‐world clinical implementation. Future studies should focus on several key strategies to overcome these challenges and enhance the effectiveness of ML‐driven models. Multi‐center validation is essential for ensuring that models generalize well across diverse populations and clinical settings, increasing confidence in their reliability and applicability [143]. Also, the integration of radiomics and genomics can provide a more comprehensive understanding of ovarian cancer, enhancing predictive capabilities by combining features from medical images with genetic data [144, 145]. Radiomics involves extracting quantitative features from medical images, such as CT scans or MRIs, which can be combined with AI algorithms to improve cancer detection and prognosis. Studies have shown that machine‐learning‐based radiomic analysis can effectively categorize ovarian tumors as benign or malignant, with high accuracy rates [146, 147].

Additionally, explainable AI techniques such as SHAP and LIME provide insights into how models make predictions, enhancing model transparency. This builds clinical trust, supports personalized medicine, and meets regulatory standards [148]. Further development of XAI techniques like LIME can improve interpretability and trustworthiness, facilitating the integration of AI models into clinical practice and improving patient outcomes [149]. Furthermore, advanced deep learning techniques, such as convolutional neural networks (CNNs) and transformer‐based models, have shown high potential in ovarian cancer detection, accelerating diagnostics and improving accuracy [150]. Finally, data augmentation methods can mitigate the impact of small sample sizes and short follow‐up periods by artificially increasing dataset sizes, reducing overfitting, and improving model reliability [151, 152]. By focusing on these strategies, future studies can move ML‐driven models closer to real‐world clinical implementation, ultimately improving early diagnosis, treatment planning, and patient outcomes.

## 7 Conclusion

The high death rate associated with ovarian cancer in women is primarily attributed to late‐stage discovery and the inadequacy of current early detection strategies. Recent advancements in ML have significantly enhanced the predictive capabilities for EOC by integrating clinical variables, imaging data, and molecular biomarkers. This review highlights the potential of ML‐driven approaches in improving diagnostic accuracy, risk stratification, and personalized treatment planning. The findings from various studies demonstrate that ML models, particularly ensemble learning methods such as RF, XGBoost, and deep learning approaches like RNNs, outperform traditional statistical methods in classifying malignant and benign ovarian tumors.

The integration of molecular biomarkers with ML techniques has significantly advanced ovarian cancer prediction, enabling improved diagnostic accuracy, risk assessment, and personalized treatment planning. This review highlights the role of key biomarkers, including CA‐125, HE4, CA72‐4, CEA, and inflammatory markers such as CRP, NLR, and LDH, in enhancing early detection and prognosis. Biomarkers such as CA‐125 and HE4 remain central to ovarian cancer prediction, with their predictive accuracy further enhanced when combined with additional biomarkers such as CEA, CA72‐4, and inflammatory markers like CRP and NLR. Furthermore, ML has proven effective in predicting treatment responses and survival outcomes by leveraging tumor burden indices, surgical complexity scores, and hematologic parameters.

ML‐based models have consistently outperformed traditional statistical approaches in ovarian cancer prediction. Ensemble methods such as RF, XGBoost, and deep learning models such as RNNs have achieved high predictive performance. Several studies reviewed in this paper demonstrated that combining tumor markers with ML techniques significantly improved classification accuracy, with reported AUC values exceeding 0.90 in models incorporating CA‐125 and HE4. Moreover, inflammatory markers (e.g., CRP, fibrinogen, and NLR) and metabolic indicators (e.g., lipid levels and blood glucose) have been successfully integrated into ML models, further enhancing early detection and treatment outcome prediction.

Despite these advancementschallenges remain in ensuring the clinical applicability of ML‐driven ovarian cancer prediction models., challenges remain in ensuring the clinical applicability of ML‐driven ovarian cancer prediction models. Variability in biomarker expression, data heterogeneity, and the need for external validation pose barriers to their widespread adoption. To achieve more precise predictive models, future studies should explore the integration of multi‐omics data, including genomics, proteomics, and metabolomics. Additionally, explainable AI techniques should be developed to enhance the interpretability of ML predictions, ensuring their seamless integration into clinical workflows. In conclusion, the combination of machine learning and biomarker‐based modeling represents a transformative approach for ovarian cancer prediction. By refining early detection strategies and optimizing risk stratification, these advancements hold the potential to improve patient outcomes and guide precision medicine initiatives in ovarian cancer management.

## Author Contributions

**Somayyeh Hormaty:** investigation (equal), conceptualization (equal), validation (equal), visualization (equal), methodology (equal), data curation (equal). **Anwar Nather Seiwan:** data curation (equal), validation (equal), visualization (equal), conceptualization (equal), investigation (equal), writing – original draft (equal), formal analysis (equal). **Bushra H. Rasheed:** conceptualization (equal), investigation (equal), writing – original draft (equal), validation (equal), visualization (equal), data curation (equal). **Hanieh Parvaz:** conceptualization (equal), visualization (equal), methodology (equal), data curation (equal), formal analysis (equal). **Ali Gharahzadeh:** investigation (equal), conceptualization (equal), writing – review and editing (equal), data curation (equal). **Hamid Ghaznavi:** conceptualization (equal), investigation (equal), writing – original draft (equal), writing – review and editing (lead), visualization (equal), validation (equal), methodology (equal), formal analysis (equal), project administration (lead), supervision (lead).

## Ethics Statement

This study is exempt from review by the ethics committee because it does not involve human participants, animal subjects, or sensitive data collection.

## Conflicts of Interest

The authors declare no conflicts of interest.

## Data Availability

Data sharing is not applicable to this article because no data sets were generated or analyzed.
